# Features of patients with rheumatoid arthritis whose debut joint is a foot or ankle joint: A 5,479-case study from the IORRA cohort

**DOI:** 10.1371/journal.pone.0202427

**Published:** 2018-09-06

**Authors:** Koichiro Yano, Katsunori Ikari, Eisuke Inoue, Yu Sakuma, Takeshi Mochizuki, Naoko Koenuma, Haruki Tobimatsu, Eiichi Tanaka, Atsuo Taniguchi, Ken Okazaki, Hisashi Yamanaka

**Affiliations:** 1 Institute of Rheumatology, Tokyo Women’s Medical University, Shinjuku, Tokyo, Japan; 2 Department of Orthopedic surgery, Tokyo Women’s Medical University, Shinjuku, Tokyo, Japan; 3 Medical Informatics, St. Marianna University School of Medicine, Kawasaki, Kanagawa, Japan; 4 Department of Orthopedic surgery, Kamagaya General Hospital, Kamagaya, Chiba, Japan; Hong Kong Polytechnic University, HONG KONG

## Abstract

**Background:**

Foot and ankle joint disorders are serious issues for patients with rheumatoid arthritis (RA). We compared the differences between patients with RA whose first symptom involved a foot or ankle joint (FOOT group) versus other joints (non-FOOT group) within the Institute of Rheumatology, Rheumatoid Arthritis (IORRA) cohort in our institute.

**Patients and methods:**

In the IORRA survey conducted in April 2016, patients were invited to complete six questionnaires about their first symptom at RA onset, current foot or ankle symptoms, daily living activities, and mental health. Disease activity, clinical laboratory variables, functional disability, quality of life, use and ratio of anti-inflammatory and antirheumatic drugs, daily living activities and mental health were compared between the two groups.

**Results:**

Among 5,637 Japanese patients with RA who participated in the IORRA survey on April 2016, 5,479 (97.2%) responded to the questionnaire regarding their debut joint. Of these patients, 2,402 (43.8%) reported that their first symptom of RA involved a foot or ankle joint. The FOOT group (n = 2,164) had higher disease activity, higher disabilities, lower quality of life, lower activities of daily living, and poorer mental health and used anti-inflammatory drugs at a higher rate and at higher doses compared with the non-FOOT group (n = 2,164). On the other hand, the use of medications to suppress the disease activity of RA was similar between the groups.

**Conclusion:**

Clinicians should pay more attention to foot and ankle joints in daily practice so as not to underestimate the disease activity of RA.

## Introduction

Foot and ankle joint disorders are serious issues for patients with rheumatoid arthritis (RA). More than 90% of patients with RA report foot symptoms at some time during the course of their disease[1, 2]. Foot and ankle joint problems in patients with RA introduce a decline in the quality of life. The most common symptoms in the foot and ankle are pain, swelling, stiffness and deformity[3]. Foot ulcers and infections resulting from deformities occasionally necessitate withdrawal of RA medications[4]. Patients with RA who have irreversible foot deformities often need orthopedic surgeries. High rates of delayed wound healing and infection complications have been associated with surgeries for rheumatoid foot[5–7], and such complications may be treated through additional drug withdrawal. In other words, foot troubles sometimes lead to exacerbation of systematic joint symptoms because of discontinuation of treatments for RA.

Rheumatoid foot is reported as the first symptom of the disease in 14%–53% of patients with RA[2, 8–12]. However, few studies have investigated the features of patients with RA whose debut joint is a foot or ankle joint. The aim of this study is to compare patients with RA whose first symptom was a foot or ankle joint versus a different joint by using the Institute of Rheumatology, Rheumatoid Arthritis (IORRA) cohort in our institute.

## Patients and methods

### IORRA cohort

The IORRA cohort study is a single-institute-based, large observational cohort of Japanese patients with RA, established in October 2000 at the Institute of Rheumatology, Tokyo Women’s Medical University. Data collection is conducted biannually. Details about the cohort study’s purpose and methodology have been previously reported[13–18]. In brief, all patients diagnosed with RA who visited our institute were registered in the IORRA cohort after informed consent was obtained. After providing informed consent, patients were invited to participate in the IORRA survey by completing a questionnaire biannually (April and October) during the period of treatment at our institute. Data were collected on each patient’s global assessment through a visual analog scale (VAS), disability using the Japanese version of the Stanford Health Assessment Questionnaire (J-HAQ)[13], and the physician’s evaluation of disease activity as measured by the swollen joint count (SJC) and tender joint count (TJC). Additional clinical laboratory variables included the erythrocyte sedimentation rate (ESR), C-reactive protein (CRP), and rheumatoid factor (RF). Patients provided information about the dosages and use of drugs, such as corticosteroids (frequency and dose converted to prednisolone equivalent), nonsteroidal anti-inflammatory drugs (NSAIDs), methotrexate (MTX), and biologics. More than 5,000 patients with RA participated in the survey, 98% of whom returned completed questionnaires by prepaid mail. The IORRA survey has been approved by the ethical committee of Tokyo Women’s Medical University (#2922-R5, #2952-R).

### Statistical analyses

In the IORRA survey conducted in April 2016, patients were invited to complete six questionnaires including their first symptom at RA onset, current foot or ankle symptoms, daily living activities and mental health (Appendix). Information on the clinical characteristics, physical examinations, laboratory tests, functional status and a measure of health status of the patients were obtained from the IORRA cohort database. Continuous data are summarized using the mean, standard deviation, and range, and categorical variables are presented as counts and percentages. All cases were classified into a FOOT group, in which the first symptom involved a foot or ankle, and the non-FOOT group, in which the first symptom did not involve a foot or ankle. Patients were matched according to age (within 5 years) and disease duration (within 2 years) at a ratio of 1:1. The Kolmogorov-Smirnov test showed that all continuous variables followed a normal distribution. A Student’s *t*-test was used to compare the following data obtained from the IORRA survey conducted in April 2016 between the FOOT and non-FOOT group: age of onset; body weight; Disease Activity Score in 28 joints calculated with ESR (DAS28); tender joint count in 28 joints (TJC28); tender joint count in 45 joints (TJC45); swollen joint count in 28 joints (SJC28); swollen joint count in 45 joints (SJC45); patient's assessment of pain on a visual analogue scale (PVAS); patient's global assessment of disease activity on a visual analogue scale (GVAS); physician's global assessment of disease activity on a visual analogue scale (DVAS); CRP; ESR; white blood cell count; RF; J-HAQ; the EuroQol 5-dimensional descriptive system (EQ-5D); prednisolone (PSL) dose per day; MTX dose per week; the difficulty in going to work, school, or shopping nearby (Q3 in Appendix); the difficulty in doing work, school activities or household duties (Q4 in Appendix); and the difficulty in taking a trip such as a business trip or journey (Q5 in Appendix). The Fisher’s exact test was used to compare categorical data, such as gender; RF positivity; anticyclic citrullinated peptide antibodies (anti-CCP) positivity on registration for the IORRA cohort study; NSAID use; PSL use; disease-modifying antirheumatic drugs (DMARDs) use; MTX use; biologic agent use; and the presence of anxiety, depression, or frustration between the two groups (Q6 in Appendix). Missing values were excluded from the analyses. Statistical analyses were performed using the R software package (http://www.r-project.org/). The level of significance was set at 0.05.

## Results

**Fig 1:**
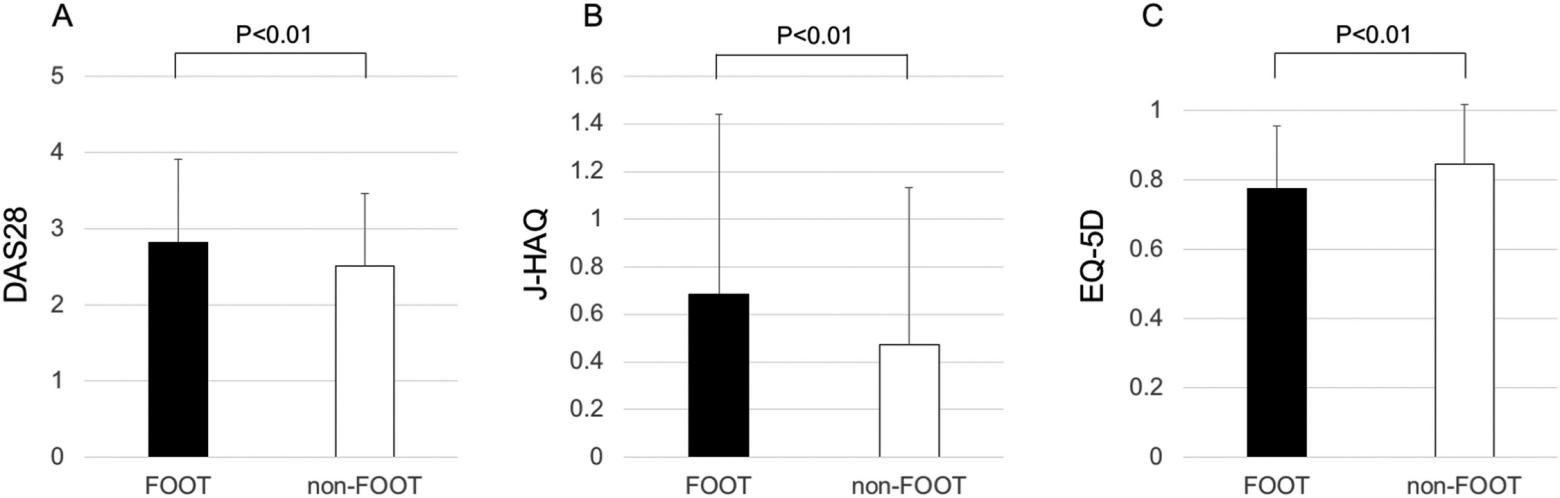
Bar graph showing the comparison of disease activity, functional disability, and quality of life between the FOOT and non-FOOT groups. A, Disease Activity Score in 28 joints calculated with ESR (DAS28). B, the Japanese version of the Stanford Health Assessment Questionnaire (J-HAQ). C, the EuroQol 5-dimensional descriptive system (EQ-5D).

**Fig 2:**
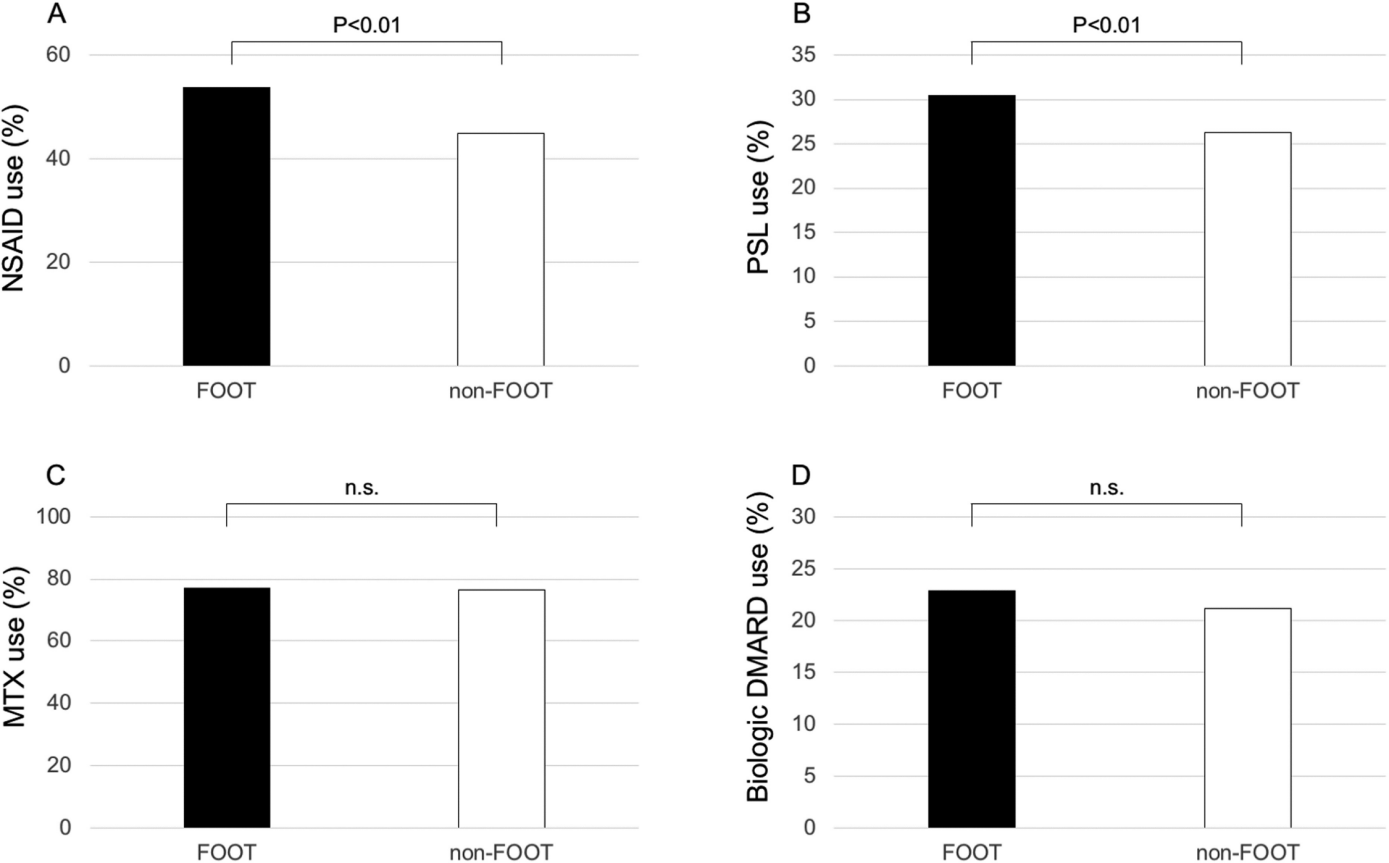
Bar graph showing the comparison of the use of drugs between the FOOT and non-FOOT groups. A, the use of nonsteroidal anti-inflammatory drugs (NSAIDs). B, the use of prednisolone (PSL). C, the use of methotrexate (MTX). D, the use of biological disease-modifying antirheumatic drugs (DMARDs). n.s. = not significant.

Among 5,637 Japanese patients with RA who participated in the IORRA survey on April 2016, 5,479 patients (97.2%) responded to the questionnaire regarding debut joint. Patient characteristics are shown in Table 1. Of these 5,479 patients, 2,402 (43.8%) reported that their first symptom of RA involved a foot or ankle. Matching for age and disease duration at a ratio of 1:1 yielded a FOOT group and a non-FOOT group that each consisted of 2,164 patients. Table 2 shows the patient characteristics in the FOOT group and the non-FOOT group. The proportion of females (87.7%) in the FOOT group was significantly higher than that (84.8%) in the non-FOOT group (*P* < .01). Body weight (53.4 ± 9.8 kg) in the non-FOOT group was significantly higher than that (52.5 ± 9.5 kg) in the non-FOOT group (*P* < .01). The proportion of patients in the FOOT group who had any current symptoms in the foot or ankle joints (71.3%) was significantly higher than that (37.3%) in the non-FOOT group (*P* < .01). To summarize the results, compared with the non-FOOT group, the patients in the FOOT group had higher disease activity (*P* < .01 for DAS28, TJC28, SJC28, PVAS, GVAS, CRP and ESR), higher seropositivity (RF, anti-CCP and RF/anti-CCP double-positive), higher disability scores (*P* < .01 for J-HAQ), lower quality of life (*P* < .01 for EQ-5D), a higher rate of use of anti-inflammatory drugs, and a higher dose of anti-inflammatory drugs (*P* < .01 for NSAID use, PSL use, and PSL dose per day) (Figs 1 and 2). On the other hand, there were no significant differences in DMARD use, MTX use, biologic agent use, and MTX dose per week between the two groups (Fig 2). Feeling difficulty in going to work, school, or shopping (Q3); in doing work, school activities or household duties (Q4); in taking a trip, such as a business trip or journey (Q5); and feeling anxious, depressed, or frustrated (Q6) were significantly more common in the FOOT group than in the non-FOOT group (*P* < .01) (Table 3).

**Table 1:** Patient characteristics (n = 5,479). *

Age, years	61.8 ± 13.0 (17–96)
Female, no. (%)	4,708 (85.9)
Disease duration, years	15.7 ± 10.2 (0–65)
DAS28	2.64 ± 1.0 (0.0–7.0)
J-HAQ	0.56 ± 0.7 (0–3)
RF, IU/mL	103.1 ± 226.7 (3–7,150)
Therapeutic drugs	
NSAID use, no. (%)	2,636 (48.1)
PSL use, no. (%)	1,516 (27.7)
PSL dose, mg/day	1.0 ± 2.1 (0–18)
MTX use, no. (%)	4,205 (76.7)
MTX dose, mg/week	6.0 ± 4.8 (0–20)
Biologic DMARD use, no. (%)	1,216 (22.2)

*Values are mean ± standard deviation (range), unless indicated otherwise. DAS28 = Disease Activity Score in 28 joints; J-HAQ = the Japanese version of the Stanford Health Assessment Questionnaire; RF = rheumatoid factor; NSAID = nonsteroidal anti-inflammatory drug; PSL = prednisolone; MTX = methotrexate; DMARD = disease-modifying antirheumatic drug.

**Table 2:** Comparison of clinical features and outcomes in the IORRA survey conducted in April 2016 between the patients whose debut joint was the foot or ankle (FOOT group) and patients whose debut joint was another joint (non-FOOT group). *

	FOOT group (n = 2,164)	non-FOOT group (n = 2,164)	P
Age, years	62.6 ± 12.3 (22–91)	62.6 ± 12.2 (21–92)	0.98
Age at onset, years	46.3 ± 12.7 (16–86)	46.4 ± 12.8 (16–84)	0.84
Disease duration, years	16.3 ± 10.3 (0–58)	16.2 ± 10.2 (0–57)	0.82
Female, no. (%)	1,898 (87.7)	1,836 (84.8)	0.007
Having any symptoms in the foot or ankle joints now, no. (%)	1,529 (71.3)^†^	800 (37.3)^‡^	<2.2e-16
Body weight, kg	52.5 ± 9.5 (27–99)	53.4 ± 9.8 (30–100)	0.004
DAS28	2.82 ± 1.1 (0.0–7.0)	2.51 ± 0.95 (0.0–6.1)	<2.2e-16
TJC28	0.7 ± 1.8 (0–24)	0.5 ± 1.1 (0–12)	1.8e-09
SJC28	1.1 ± 2.1 (0–18)	0.7 ± 1.6 (0–18)	2.0e-13
TJC45	1.1 ± 2.6 (0–24)	0.6 ± 1.4 (0–16)	<2.2e-16
SJC45	1.3 ± 2.5 (0–30)	0.8 ± 1.7 (0–18)	1.7e-15
PVAS, mm	25.8 ± 24.7 (0–100)	17.5 ± 20.8 (0–100)	<2.2e-16
GVAS, mm	29.3 ± 24.9 (0–99)	20.9 ± 21.9 (0–100)	<2.2e-16
DVAS, mm	10.4 ± 13.0 (0–83)	7.3 ± 9.8 (0–82)	<2.2e-16
CRP, mg/dL	0.44 ± 0.92 (0.0–9.7)	0.32 ± 0.80 (0.0–18.3)	4.1e-06
ESR, mm/h	24.6 ± 19.3 (1.0–100.0)	21.6 ± 16.8 (1.0–100.0)	1.0e-07
WBC, /μL	6018.5 ± 1973.7	5798.2 ± 1948.1	0.0002
	(2170–16820)	(2000–39880)	
RF, IU/mL	118.5 ± 260.7 (3–7150)	97.2 ± 217.1 (3–5544)	0.004
RF positivity, no (%)	1,824 (77.2)	2,178 (71.0)	2.9e-07
anti-CCP positivity, no (%)	1,795 (86.3)	2,218 (80.3)	4.1e-08
RF/anti-CCP double-positive, no (%)	1,518 (73.5)	1,795 (65.5)	2.5e-09
J-HAQ	0.69 ± 0.76 (0–3)	0.47 ± 0.66 (0–3)	<2.2e-16
EQ-5D	0.78 ± 0.18 (-0.1–1.0)	0.85 ± 0.2 (0.1–1.0)	<2.2e-16
NSAID use, no. (%)	1,162 (53.7)	972 (44.9)	8.9e-09
PSL use, no. (%)	659 (30.5)	569 (26.3)	0.003
PSL dose, mg/day	1.17 ± 2.3 (0–18)	0.9 ± 2.0 (0–15)	0.0001
DMARD use, no. (%)	1,926 (89.0)	1,921 (88.8)	0.85
MTX use, no. (%)	1,671 (77.2)	1,656 (76.5)	0.61
MTX dose, mg/week	6.18 ± 4.8 (0–17.5)	5.88 ± 4.7 (0–20)	0.059
Biologic DMARD use, no. (%)	495 (22.9)	458 (21.2)	0.19

* Values are mean ± standard deviation (range), unless indicated otherwise.

DAS28 = Disease Activity Score in 28 joints; TJC28 = tender joint count in 28 joints; SJC28 = swollen joint count in 28 joints; TJC45 = tender joint count in 45 joints; SJC45 = swollen joint count in 45 joints; PVAS = patient's assessment of pain on a visual analogue scale; GVAS = patient's global assessment of disease activity on a visual analogue scale; DVAS = physician's global assessment of disease activity on a visual analogue scale; CRP = C-reactive protein; ESR = erythrocyte sedimentation rate; WBC = white blood cell count; RF = rheumatoid factor; anti-CCP = anticyclic citrullinated peptide antibodies; J-HAQ = the Japanese version of the Stanford Health Assessment Questionnaire; EQ-5D = the EuroQol 5-dimensional descriptive system; NSAID = nonsteroidal anti-inflammatory drug; PSL = prednisolone; DMARD = disease-modifying antirheumatic drug; MTX = methotrexate.

^†^ Missing data for having current foot or ankle symptoms in the FOOT group (n = 19).

^‡^ Missing data for having current foot or ankle symptoms in the non-FOOT group (n = 20).

**Table 3:** Comparison of daily living activities and mental health in the IORRA survey conducted in April 2016 between the patients whose debut joint was the foot or ankle (FOOT group) and patients whose debut joint was another joint (non-FOOT group). *

		FOOT group (n = 2,164)		non-FOOT group (n = 2,164)		P	
Difficulty in going to work, school, or shopping nearby^†^		0.60 ± 0.76 (0–3)		0.28 ± 0.59 (0–3)		<2.2e-16	
Difficulty in doing work, school activities or household duties^†^		0.55 ± 0.75 (0–3)		0.26 ± 0.57 (0–3)		<2.2e-16	
Difficulty in taking a trip, such as a business trip or journey^†^		0.69 ± 0.83 (0–3)		0.36 ± 0.66 (0–3)		<2.2e-16	
Feeling anxious, depressed, or frustrated, positivity, no. (%)^‡^		932 (43.4)^§^		441 (20.7)^¶^		<2.2e-16	

* Values are mean ± standard deviation (range), unless indicated otherwise.

^†^The following response categories are available for each question: without any difficulty (score 0), with low difficulty (score 1), with moderate difficulty (score 2), or severe difficulty (score 3).

^‡^Patients answered with Yes or No.

^§^ Missing data for feeling anxious, depressed, or frustrated in the FOOT group (n = 18).

^¶^ Missing data for feeling anxious, depressed, or frustrated in the non-FOOT group (n = 35).

## Discussion

Measures of disease activity utilizing 28-joint counts in RA, such as DAS28, have been validated for assessment of minimal disease activity and remission in clinical trials[19–21]. However, since 28-joint count assessments do not include examination of the foot and ankle, physicians tend to omit examinations of feet and ankles in routine practice. Indeed, Souza et al. reported that 54% of clinicians did not examine feet routinely because they are not included in the DAS28[22]. Also, physicians may tend to give priority to a quick hand examination over a time-consuming foot and ankle examination within tight time constraints in daily practice. Otter et al. reported that the last foot examination of respondents in their study was 16.5 months ago, whereas the last hand examination was 6.2 months ago[11]. However, there are some reports that foot synovitis was detected in a substantial proportion of patients classified as being in clinical remission according to DAS28[23, 24]. Though examinations of 28 joints may be enough to assess the disease activity in large-scale studies, physicians should pay attention to more joints, including foot and ankle joints, in daily practice.

In this single-institute–based, large observational cohort of 5,479 patients with RA, 43.8% of respondents indicated that the foot or ankle was the first site involved. In other studies investigating more than 500 RA patients, debut in the foot or ankle has been reported in 20%–53%, corresponding well with our results[2, 10, 11]. The respondents in this study were divided into two groups according to the debut joint, foot/ankle or others, after matching for age and disease duration. Among the two groups, the proportions using medications to suppress the disease activity of RA were not statistically different. However, the DAS28 score in the foot or ankle onset group was significantly higher than that in the other onset group, even though the foot and ankle were not included in the 28 joints for assessing the disease activity. Not only the TJC28 and SJC28 scores, which omit examinations of the foot and ankle, but also the VAS and laboratory data associated with inflammation were higher in the foot or ankle onset group. Furthermore, a higher proportion of that group was found to use anti-inflammation drugs. Also, the FOOT group felt more difficulties in daily living activities and mental health, compared with the non-FOOT group. To summarize the results of this study, patients whose debut joint was a foot or ankle had higher disease activity, higher seropositivity, higher disabilities, lower quality of life, lower activities of daily living, and poorer mental health and used anti-inflammatory drugs at a higher rate as well as at higher doses, compared with the non-FOOT group. Considering that the proportion of patients in the FOOT group currently having any foot or ankle symptoms was significantly higher than that in the non-FOOT group, the underestimation in disease activity from omitting the examination of foot and ankle joints may lead to inadequate control of RA as stated in the past reports[24, 25]. Therefore, it may be naturally more difficult to control the disease activity in patients with RA whose debut joint was the foot or ankle. RA from foot and ankle joints may have a different pathology from that of other joints. Future studies are required to verify this hypothesis.

This study has several limitations. First, IORRA is a single institute based cohort study, so the results may not be generalizable to all patients with RA. Second, the proportion of females (85.9%) in this study is higher than in previous reports[26]. Consequently, foot or ankle symptoms as debut joints may include foot deformities unrelated to RA, such as hallux valgus, bunionette, and flatfoot because the toes/forefoot are the most common anatomical sites of pain in women[27]. These deformities occurring before RA onset might be mistakenly regarded as RA debut joints.

In conclusion, patients whose debut joint was a foot or ankle had higher disease activity, higher seropositivity, higher dysfunction, lower quality of life, lower activities of daily living, and more foot or ankle symptoms now, were more likely to use anti-inflammatory drugs, and used a higher dose of anti-inflammatory drugs than patients whose debut joint was another joint at RA onset in a present cross-sectional study. Clinicians should pay more attention to foot and ankle joints in daily practice so as not to underestimate the disease activity of RA.

## Appendix: Questionnaires

(Q1) Did your first symptoms (eg, pain, swelling, and deformity) of RA occur in the foot or ankle joints?
(Q2) Do you have any symptoms (eg, pain, swelling, and deformity) in the foot or ankle joints now?
(Q3) Do you feel difficulty in going to work, school, or shopping nearby because of your foot or ankle symptoms?
(Q4) Do you feel difficulty in doing work, school activities or household duties because of your foot or ankle symptoms?
(Q5) Do you feel difficulty in taking a trip, such as a business trip or journey because of your foot or ankle symptoms?
(Q6) Do you feel anxious, depressed, or frustrated because of your foot or ankle symptoms?

The questions from Q3 to Q6 were extracted from self-administered foot-evaluation questionnaire (SAFE-Q)[28]. In Q1, Q2 and Q6, patients answered with Yes or No. From Q3 to Q5, the following response categories are available for each question: without any difficulty (score 0), with low difficulty (score 1), with moderate difficulty (score 2), or severe difficulty (score 3).
